# Supplementary material for: Conditional chemoconnectomics (cCCTomics) as a strategy for efficient and conditional targeting of chemical transmission
Source: eLife. 2024 Apr 30;12:RP91927. doi: 10.7554/eLife.91927 (PMC11060718; doi:10.7554/eLife.91927)
Supplement: Figure 3—source data 3. [file elife-91927-fig3-data3.zip › Figure 3 source data3 part 1.zip!/Figure 3-Source data 3-part1/1175-9-CRISPResso_Report_rtnd62ko.zip!/CRISPResso_on_1175_12/9.Alleles_frequency_table_around_sgRNA_gtaggccacgcagtagacca.pdf]

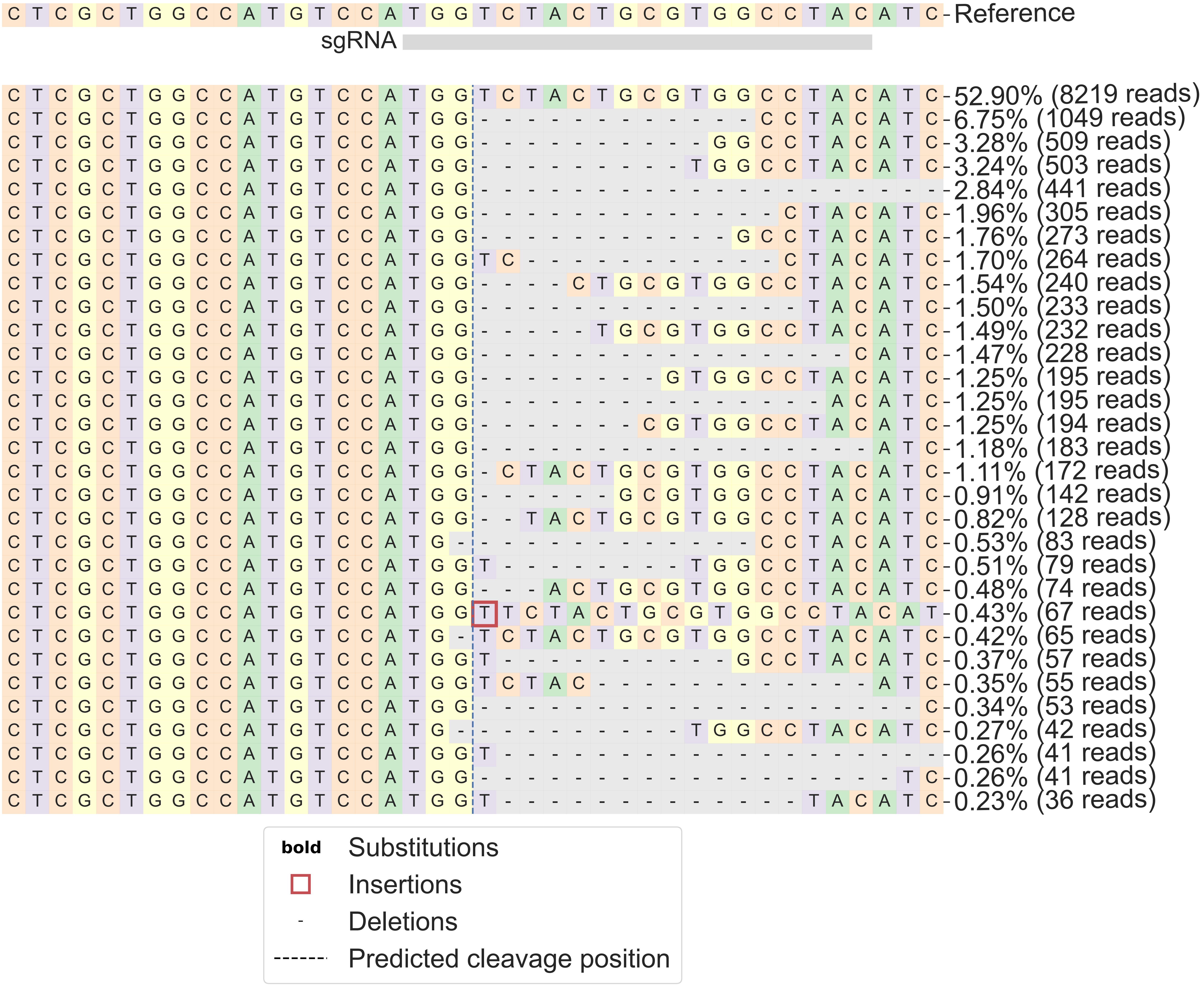
Reference
sgRNA
52.90% (8219 reads)
6.75% (1049 reads)
3.28% (509 reads)
3.24% (503 reads)
2.84% (441 reads)
1.96% (305 reads)
1.76% (273 reads)
1.70% (264 reads)
1.54% (240 reads)
1.50% (233 reads)
1.49% (232 reads)
1.47% (228 reads)
1.25% (195 reads)
1.25% (195 reads)
1.25% (194 reads)
1.18% (183 reads)
1.11% (172 reads)
0.91% (142 reads)
0.82% (128 reads)
0.53% (83 reads)
0.51% (79 reads)
0.48% (74 reads)
0.43% (67 reads)
0.42% (65 reads)
0.37% (57 reads)
0.35% (55 reads)
0.34% (53 reads)
0.27% (42 reads)
0.26% (41 reads)
0.26% (41 reads)
0.23% (36 reads)
bold Substitutions
Insertions
Deletions
Predicted cleavage position
